# Supplementary figures and images for: The effect of a hiding box on stress levels and body weight in Dutch shelter cats; a randomized controlled trial
Source: PLoS One. 2019 Oct 14;14(10):e0223492. doi: 10.1371/journal.pone.0223492 (PMC6791553; doi:10.1371/journal.pone.0223492)

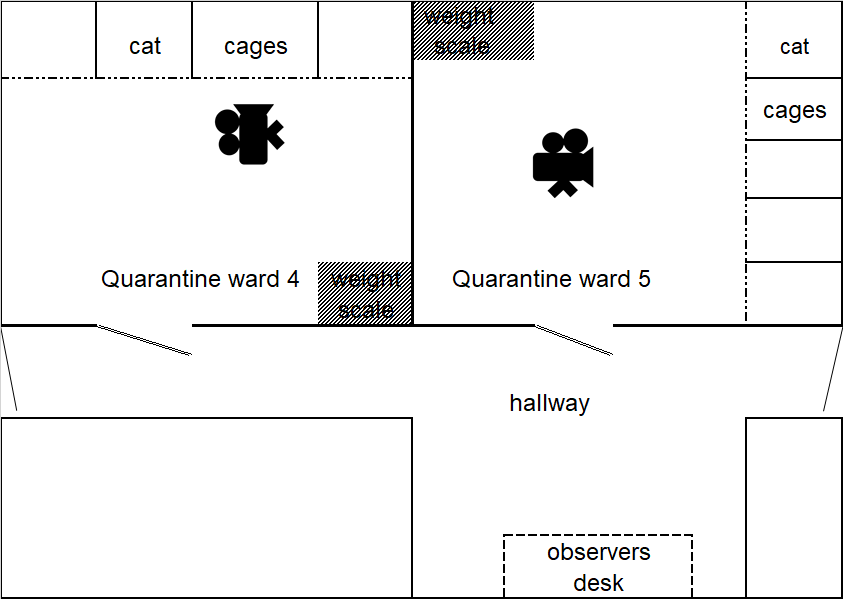

Supplement: S1 Appendix — (BMP) [file pone.0223492.s001.bmp]

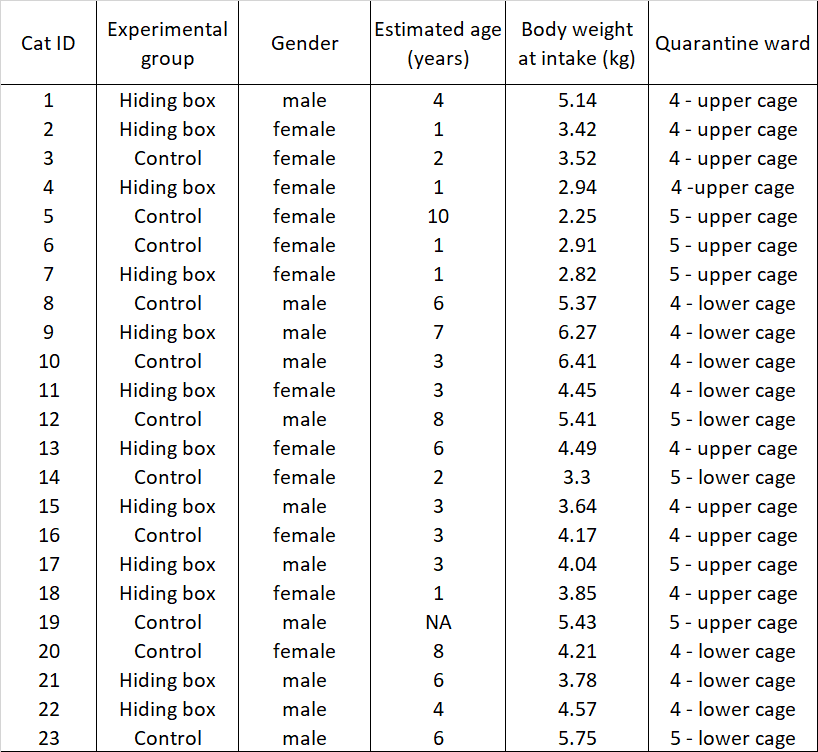

Supplement: S2 Appendix — (BMP) [file pone.0223492.s002.bmp]
