# Supplementary material for: The effect of a hiding box on stress levels and body weight in Dutch shelter cats; a randomized controlled trial
Source: PLoS One. 2019 Oct 14;14(10):e0223492. doi: 10.1371/journal.pone.0223492 (PMC6791553; doi:10.1371/journal.pone.0223492)
Supplement: Attachment — Submitted filename: PONE-D-19-15024_reviewer(1).pdf [file pone.0223492.s003.pdf]

PLOS ONE

The effect of a hiding box on stress levels and body weight in Dutch shelter cats; a randomized controlled trial

--Manuscript Draft--

**Manuscript Number:**	PONE-D-19-15024
**Article Type:**	Research Article
**Full Title:**	The effect of a hiding box on stress levels and body weight in Dutch shelter cats; a randomized controlled trial
**Short Title:**	Effect of hiding box on stress levels and body weight in Dutch shelter cats; a randomized controlled trial
**Corresponding Author:**	Ruth van der Leij<br>Universiteit Utrecht Faculteit Diergeneeskunde<br>Utrecht, NETHERLANDS
**Keywords:**	
**Abstract:**	While staying in an animal shelter, cats may suffer from chronic stress which impairs their health and welfare. Offering hiding opportunities can significantly reduce behavioural stress in cats, but confirmation with physical parameters is needed. Therefore, the aim of this study was to determine the effect of a hiding box on behavioural stress levels (scored by means of the Cat-Stress-Score) and a physical parameter as body weight in newly arrived cats in a Dutch animal shelter during the first 12 days in quarantine situations.<br>Twenty three cats between 1 and 10 years of age were randomly divided between the experimental (N = 12) and control group (N = 11) with and without a hiding box. Stress levels were assessed on days 1, 2, 3, 5, 7, 9 and 12 according to the non-invasive Cat-Stress-Score (CSS). Body weights were measured on days 0, 7 and 12. Finally, adoption rates and length of stay (LOS) were determined.<br>Major findings of the study are: (1) the mean Cat-Stress-Score decreased with time for all cats, but cats with a hiding box however showed a significant faster decrease in the CSS, reaching a lower CSS-steady state seven days earlier than the control group; (2) nearly all cats in both groups lost significant body weight during the first two weeks; (3) hiding boxes did not significantly influence weight loss; (4) no differences were found in the adoption rates and the LOS between both groups.<br>Hiding enrichment reduces behavioural stress in shelter cats during quarantine situations and can therefore be a relatively simple aid to shelter adaptation. It offers no prevention however against feline weight loss, which indicates a serious health risk for shelter cats.
**Order of Authors:**	Ruth van der Leij
	Lara Dorothea Alberta Maria Selman
	Johannes C.M. Vernooij
	Claudia Maureen Vinke
**Additional Information:**	
**Question**	**Response**
**Financial Disclosure**<br><br>Enter a financial disclosure statement that describes the sources of funding for the work included in this submission. Review the submission guidelines for detailed requirements. View published research articles from *PLOS ONE* for specific examples.	W.J.R. van der Leij, the corresponding author of this manuscript, received financial support for this study from the Maria Naundorf Van Gorkum Fund (grant number: DG.023003) and from Mrs. R. Brons.<br>We thank them for their generous support of this research.<br>Fund Maria Naundorf Van Gorkum: https://www.uu.nl/en/organisation/alumni/well-spent/named-funds<br>The funders had no role in study design, data collection and analysis, decision to publish, or preparation of the manuscript.

Powered by Editorial Manager® and ProduXion Manager® from Aries Systems Corporation

This statement is required for submission and **will appear in the published article** if the submission is accepted. Please make sure it is accurate.<br><br>**Unfunded studies**<br>Enter: *The author(s) received no specific funding for this work.*<br><br>**Funded studies**<br>Enter a statement with the following details:<br>• Initials of the authors who received each award<br>• Grant numbers awarded to each author<br>• The full name of each funder<br>• URL of each funder website<br>• Did the sponsors or funders play any role in the study design, data collection and analysis, decision to publish, or preparation of the manuscript?<br>• **NO** - Include this sentence at the end of your statement: *The funders had no role in study design, data collection and analysis, decision to publish, or preparation of the manuscript.*<br>• **YES** - Specify the role(s) played.<br><br>* typeset	
**Competing Interests**<br><br>Use the instructions below to enter a competing interest statement for this submission. On behalf of all authors, disclose any competing interests that could be perceived to bias this work—acknowledging all financial support and any other relevant financial or non-financial competing interests.<br><br>This statement **will appear in the published article** if the submission is accepted. Please make sure it is accurate. View published research articles from *PLOS ONE* for specific examples.	The authors have declared that no competing interests exist.

Powered by Editorial Manager® and ProduXion Manager® from Aries Systems Corporation

**NO authors have competing interests**<br><br>Enter: *The authors have declared that no competing interests exist.*<br><br>**Authors with competing interests**<br><br>Enter competing interest details beginning with this statement:<br><br>*I have read the journal's policy and the authors of this manuscript have the following competing interests: [insert competing interests here]*<br><br>* typeset	
**Ethics Statement**<br><br>Enter an ethics statement for this submission. This statement is required if the study involved:<br><br>• Human participants<br>• Human specimens or tissue<br>• Vertebrate animals or cephalopods<br>• Vertebrate embryos or tissues<br>• Field research<br><br>Write "N/A" if the submission does not require an ethics statement.<br><br>General guidance is provided below. Consult the submission guidelines for detailed instructions. **Make sure that all information entered here is included in the Methods section of the manuscript.**	The study was approved by the Animal Welfare Body Utrecht, after assessing the present study. It was concluded that the study does not meet the definition of an animal experiment as defined in the Dutch Experiments on Animals Act and Directive 2010/63/EU because the animals encountered no discomfort.<br>Name of the institution that granted permission: Animal Welfare Body Utrecht, https://www.uu.nl/en/news/animal-welfare-body-utrecht

Powered by Editorial Manager® and ProduXion Manager® from Aries Systems Corporation

**Format for specific study types**<br><br>**Human Subject Research (involving human participants and/or tissue)**<br>• Give the name of the institutional review board or ethics committee that approved the study<br>• Include the approval number and/or a statement indicating approval of this research<br>• Indicate the form of consent obtained (written/oral) or the reason that consent was not obtained (e.g. the data were analyzed anonymously)<br><br>**Animal Research (involving vertebrate animals, embryos or tissues)**<br>• Provide the name of the Institutional Animal Care and Use Committee (IACUC) or other relevant ethics board that reviewed the study protocol, and indicate whether they approved this research or granted a formal waiver of ethical approval<br>• Include an approval number if one was obtained<br>• If the study involved *non-human primates*, add *additional details* about animal welfare and steps taken to ameliorate suffering<br>• If anesthesia, euthanasia, or any kind of animal sacrifice is part of the study, include briefly which substances and/or methods were applied<br><br>**Field Research**<br><br>Include the following details if this study involves the collection of plant, animal, or other materials from a natural setting:<br>• Field permit number<br>• Name of the institution or relevant body that granted permission	
**Data Availability**<br><br>Authors are required to make all data underlying the findings described fully available, without restriction, and from the time of publication. PLOS allows rare exceptions to address legal and ethical concerns. See the PLOS Data Policy and FAQ for detailed information.	Yes - all data are fully available without restriction

A Data Availability Statement describing where the data can be found is required at submission. Your answers to this question constitute the Data Availability Statement and **will be published in the article**, if accepted.<br><br>**Important:** Stating 'data available on request from the author' is not sufficient. If your data are only available upon request, select 'No' for the first question and explain your exceptional situation in the text box.<br><br>Do the authors confirm that all data underlying the findings described in their manuscript are fully available without restriction?	
**Describe where the data may be found in full sentences. If you are copying our sample text, replace any instances of XXX with the appropriate details.**<br><br>• If the data are **held or will be held in a public repository**, include URLs, accession numbers or DOIs. If this information will only be available after acceptance, indicate this by ticking the box below. For example: *All XXX files are available from the XXX database (accession number(s) XXX, XXX.*).<br>• If the data are all contained **within the manuscript and/or Supporting Information files**, enter the following: *All relevant data are within the manuscript and its Supporting Information files.*<br>• If neither of these applies but you are able to provide **details of access elsewhere**, with or without limitations, please do so. For example:<br><br>*Data cannot be shared publicly because of [XXX]. Data are available from the XXX Institutional Data Access / Ethics Committee (contact via XXX) for researchers who meet the criteria for access to confidential data.*<br><br>*The data underlying the results presented in the study are available from (include the name of the third party*	The data are placed in a public repository, named DataVERSE. Some minor revisions still need to be made after which a DOI will be issued for this dataset.

and contact information or URL).<br>• This text is appropriate if the data are owned by a third party and authors do not have permission to share the data.<br><br>* typeset	
Additional data availability information:	

Manuscript

Click here to download Manuscript Artikel Plos One - 17 mei 2019.docx

The effect of a hiding box on stress levels and body weight in Dutch shelter cats; a randomized controlled trial

Wilhelmina Johanna Ruth van der Leij (ORCID 0000-0002-0164-8007) [1,¶,#a,*], Lara Dorothea Alberta Maria Selman [2,4¶], Johannes C.M. Vernooij (ORCID 0000-0002-2646-9216)[3,#b], Claudia Maureen Vinke [4,#c]

[1] Department of Clinical Sciences of Companion Animals, Shelter Medicine Program, Faculty of Veterinary Medicine, Utrecht University, The Netherlands

[2] Department of Clinical Sciences of Companion Animals, Faculty of Veterinary Medicine, Utrecht University, The Netherlands

[3] Department of Farm Animal Health, Faculty of Veterinary Medicine, Utrecht University, The Netherlands

[4] Department of Animals in Science and Society, Faculty of Veterinary Medicine, Utrecht University, The Netherlands

[#a] Current Address: Department of Clinical Sciences of Companion Animals, Faculty of Veterinary Medicine, Utrecht University, Yalelaan 108, PO Box 80.154, 3508 TD, Utrecht, The Netherlands

[#b] Current Address: Department of Farm Animal Health, Faculty of Veterinary Medicine, Utrecht University, Yalelaan 7, 3584 CL Utrecht, The Netherlands

[#c] Current Address: Department of Animals in Science and Society, Faculty of Veterinary Medicine, Utrecht University, Yalelaan 2, PO Box 80166, 3508 TD Utrecht, The Netherlands

* Corresponding author

E-mail: W.J.R.vanderleij@uu.nl

¶ These authors contributed equally to this work.

Abstract

While staying in an animal shelter, cats may suffer from chronic stress which impairs their health and welfare. Offering hiding opportunities can significantly reduce behavioural stress in cats, but confirmation with physical parameters is needed. Therefore, the aim of this study was to determine the effect of a hiding box on behavioural stress levels (scored by means of the Cat-Stress-Score) and a physical parameter as body weight in newly arrived cats in a Dutch animal shelter during the first 12 days in quarantine situations.

Twenty three cats between 1 and 10 years of age were randomly divided between the experimental (N = 12) and control group (N = 11) with and without a hiding box. Stress levels were assessed on days 1, 2, 3, 5, 7, 9 and 12 according to the non-invasive Cat-Stress-Score (CSS). Body weights were measured on days 0, 7 and 12. Finally, adoption rates and length of stay (LOS) were determined.

Major findings of the study are: (1) the mean Cat-Stress-Score decreased with time for all cats, but cats with a hiding box however showed a significant faster decrease in the CSS, reaching a lower CSS-steady state seven days earlier than the control group; (2) nearly all cats in both groups lost significant body weight during the first two weeks; (3) hiding boxes did not significantly influence weight loss; (4) no differences were found in the adoption rates and the LOS between both groups.

Hiding enrichment reduces behavioural stress in shelter cats during quarantine situations and can therefore be a relatively simple aid to shelter adaptation. It offers no prevention however against feline weight loss, which indicates a serious health risk for shelter cats.

Introduction

About 200 animal shelters in the Netherlands take in and rehome 27.000 stray and relinquished cats annually [1]. A shelter life is often associated with many stressors. Cats entering a shelter are introduced to a foreign environment with unfamiliar animals, people, sounds and smells. During these first days many of these cats struggle to adapt to these prolonged or repeated stressors and thus show stress responses [2-4]. This may elicit clinical signs like hiding behaviour, defecating and urinating outside the litter box, decreased grooming or over-grooming behaviour and a loss of appetite [2,5-8]. Stress-induced longterm high cortisol levels can reduce the efficacy of the immune system against infectious diseases [1,5,6,8,9], and chronic stress can therefore harm a cat's health as well [5,7,10,11].

When in a state of stress, the majority of cats will stop eating. Tanaka et al. found that stress elicited a decrease in food intake, negatively correlated with stress scores [12,13]. This stress response can have grave impact on cats: severe body weight losses in only a short period of time can induce feline hepatic lipidosis [5,14,15].

Several studies show that stressed cats display increased alert resting behaviour behind their litter box in an environment without hiding opportunities [10,16,17]. This is interpreted as alternative hiding behaviour for it offers some concealment [10,16]. Real concealment can be offered by providing a hiding box to shelter cats. A study of Kry and Casey [17] demonstrated a decrease in stress, measured by the Cat-Stress-Score (CSS), when shelter cats were offered hiding boxes. Weight loss during quarantine is another phenomenon in shelter cats associated with stress [12]. However, little research has been done on the preventive effect of a hiding box on this stress induced weight loss.

A previous study conducted by Vinke et al. has been the first step to scientific evidence about the effect of a hiding box on stress levels of newly arrived cats in a Dutch animal shelter during the first 14 days in quarantine situations. The results show that cats with a hiding box recovered at least 4 days earlier from stress than cats without a hiding box [10]. The present study was designed with more frequent CSS scoring between day 5 and 12, to gain more insight in the feline recovery to stress and to relate these behavioural stress levels to a physical parameter as body weight.

The primary aim of this study was to determine the effect of a hiding box on behavioural stress levels and on body weight of newly arrived cats in a Dutch animal shelter during the first 12 days in quarantine. The additional aim was to compare the Length of Stay (LOS) of cats in both study groups. It was hypothesized that a hiding box would significantly reduce stress levels of newly arrived cats compared to the non-hiding box group, reflected in a lower CSS, less weight loss and a shorter LOS.

Materials and methods

The study was approved by the Animal Welfare Body Utrecht, after assessing the present study. It was concluded that the study does not meet the definition of an animal experiment as defined in the Dutch Experiments on Animals Act and Directive 2010/63/EU because the animals encountered no discomfort.

Animal shelter

This study was performed at a Dutch animal shelter (Stichting Dierentehuis Arnhem en omstreken), a medium size animal shelter with an open intake of around 700 cats per year [31]. Cat housing is situated in five separate quarantine units, an isolation ward and an adoption unit, providing a maximum shelter capacity of 90 cats in total. Dutch legislation mandates animal shelters to have quarantine and isolation wards and a legal stray holding period of 14 days. New animals were quarantined at intake for at least 2 weeks, as is legally required [19]. For this study an informed consent was obtained from the shelter staff. In order to relate this study to daily shelter management, the original shelter protocols about the intake of new animals, daily animal care and hygiene were generally accepted, and substantial adjustments were avoided.

Animals

For this study 23 European short hair cats, 11 males and 12 females, were selected out of the cats entering the shelter between the 4th of November and the 30th of December 2015. At intake cats entering the shelter were examined by the shelter staff for gender, breed and age and received a treatment against

ecto- and endoparasites (Stronghold® and Milbemax®). As all the cats came in as strays, age was estimated in years. Within 5 days after intake the shelter veterinarian performed a physical health check. During this veterinary check (during the morning hours) the cats were microchipped and vaccinated with an attenuated vaccin (Versifel CVR®) against feline panleukopenia virus (FPV), feline herpes virus (FHV-1) and feline calici virus (FCV). Intact cats were spayed or neutered after Day 14.

Inclusion criteria for this study were based on breed (European shorthair cats), health status and age (between 1 and 10 years of age). When new cats showed no clinical signs of illness, obvious heat, pregnancy or signs of nursing during the physical examination at intake, they were included in this study. As it is not generally accepted practice in Dutch animal shelters to screen apparently healthy cats through diagnostic testing (e.g. FIV/FeLV) at shelter intake, apart from the physical examination, no additional information was available on the feline health status of the cats in this study.

All cats were observed for at least 12 days after intake.

During the study two cats participating in this study, left the shelter before their last observation day: from the Hiding box group one cat went to a foster home, from the Control group one cat was released within a trap-neuter-relaese (TNR) program. Data of both cats were excluded from this study. Two other cats were not included in data for the length of stay, but were included in data for the Cat-Stress-Score, body weigth and the adoption rate. After the 12 days observation period, one of these cats (nr. 8, control group) proved to be infected with FeLV and was euthanized a few days after the quarantine period of 14 days, while another cat (nr. 19, control group), because of its semi feral behaviour, was also released through the TNR program. Because shelters often take in these non-clinical but infected cats and stray cats being poorly socialized (and even rehome them), this study has included these two cats in three of the four measured parameters.

The reason for selecting cats between 1 and 10 years of age was to avoid inclusion of juveniles with related specific behaviour and elderly cats with increased chances for subclinical disease which might influence their behaviour [18,20]. As previous studies [17,18] found no gender related significant differences in stress behaviour, both male and female cats were included in the present study. The 23 cats were randomly assigned to one of the two groups with and without access to a hiding box.

Housing conditions

The cat housing in the two adjacent quarantine wards consisted of cages (L x W x H: either 84 x 95 x 80 cm or 69 x 91 x 87 cm) in which the cats were individually housed. Every cage was furnished with a food and water bowl, bedding of towels, a litter box and a perching shelf at 28 cm above the cage floor. The cages of the experimental group contained a hiding box that was placed at the right side at the back of the cage. To avoid place preference for towels as bedding, the towels were covering the entire floor of the cage including the shelf and the inside of the hiding box.

Cardboard boxes were used as hiding box and measured 44 x 31 x 26 cm (L x W x H). These boxes had two entrances (WxH 0.16 x 0.20 m) [10]. Hiding boxes were never reused.

Access to the cats in the quarantine wards was restricted to the caretakers and the observer. Natural daylight was provided through windows in both quarantine wards, combined with fluorescent lighting between 08:00 AM. and 5:00 PM. Daily temperatures in the quarantine wards ranged from 16.0 to 19.8 °C. In the quarantine wards no dog vocalizations could be heard.

Daily animal care

The shelter staff cleaned the cages daily between 09:00 and 12:15 AM by removing waste and applying a spot cleaning method [21]. During this procedure cats remained in their cages. Litter boxes were daily cleaned with hot water and dried with clean paper towels. Cages were disinfected between cats or when indicated (e.g. diarrhea) with a chlorine disinfectant containing sodium dichloroisocyanurate (Halacid®).

Food was provided once daily between 9:30 and 10:00 AM and comprised of around 50g per day Adult Royal Canin® dry cat food (SC 365D) with a metabolizable energy content (ME) of 4066 kcal/kg (16.995 MJ/kg). Fresh water was provided ad libitum. Cats kept their own litter box for the time of this study.

Behavioural observations

Cats were given an habituation period of 24 hours after shelter intake (= Day 0), before behavioural assessment was performed [17]. Behavioural data were collected on days 1, 2, 3, 5, 7, 9 and 12 between 12:30 and 5:15 PM, during which interactions with caretakers were avoided.

Each cat was observed for 20 minutes per day by using video-recording. Outside the cage a video camera (H.264 DVR) was mounted on a tripod at cage height. For new observations the combination camera-tripod had to be readjusted to the new cat cage. Video recordings were viewed in real-time in an adjacent room and stored for subsequent analysis (Fig 1). Only one camera was used for recording.

Fig 1. Diagram of the experimental set up, observer and both camera positions in the quarantine wards in the animal shelter.

Cat-Stress-Score (CSS)

Kessler and Turner [18,22] developed a 7-level cat stress score (CSS) which has been used in several studies to estimate stress levels in confined cats [3,10,17,18,]. This scoring system assesses the level of feline stress based on the posture of body elements (e.g. belly, legs, tail, head, eyes, pupils, ears, whiskers) and behaviour (vocalization and activity) as described in the ethogram of the UK Cat Behaviour Working Group [22]. The CSS ranges from 1 (fully relaxed) to 7 (terrorized).

One observer (LS) assessed the CSS score per cat on Day 1, 2, 3, 5, 7, 9 and 12. Intra-observer variation was minimized by observational training using (video) images of pre-described feline behaviours from previous experiments with shelter cats.

After the video camera had been positioned, the scan sampling started after 2 minutes in which the cat habituated to the novel situation. Thereafter the cat was scored according to the Scan Sampling method, in which four scores (= four samplings) were made during the observation time (the 1st observation at 5 min, the 2nd at 10 min, the 3rd at 15 min and the 4th at 20 min) [23]. Imperceptible posture and behavioural elements were noted as missing values.

Each of the elements of the Cat-Stress-Scores was scored separately.The scores of the four samplings were averaged to assign an overall CSS for each seperate cat per day.

Body weight

During the study every cat was weighted on Day 0, 7 and 12 by using an electronic scale[a] (accuracy ± 10 g). The standardized shelter feeding regime consisted of approximately 50g per cat per day of Adult Royal Canin (RC)® dry cat food, which equals 203.3 kcal or 849.8 kJ per cat per day.

To secure adequate nutrition for the cats in this study, the daily caloric feline requirements (FEDIAF guidelines (80 kcal [335 kJ] ME per kg0.67)) were determined per individual cat [24].

Adoption rates and length of stay (LOS)

In order to determine the effect of a hiding box in quarantine situations on the subsequent adoption success, the adoption dates of the cats in this study were noted. Adoption rates (= # cats adopted / all cats in this study) and the length of stay (LOS: number of days between the shelter intake of a cat and its day of adoption) was determined per cat. The LOS included the mandatory quarantine period of two weeks and only included adopted cats, excluding cats which were euthanized or returned to their outdoor environment after finishing this study.

Statistical analyses

A randomised controlled trial (RCT) design was used [25]. Data were stored in Microsoft Excel 2010 files (Microsoft Corp, Redmond, Wash.). Two statistical software programs were used for analysis of the data:

- SPSS (IBM Corp, Armonk, NY version 25) for the two-sample T-test and chi-square test.
- R (version 3.3.0) for the linear mixed regression models [28].

For the statistical analysis of effect of time and hiding box on the CSS (model ‘CSS-Time-Box’) a linear mixed regression model [27] was assumed, with the CSS as the outcome, while Time after arrival, the availability of a hiding box and the interaction between both were used as explanatory factors. CatID was used as the random effect to take the correlation between observations within cat into account. An AR1 correlation between the time points was added as well as a variance model to allow different variances for the separate time points. A maximum likelihood-based method was used to calculate the

Akaike's Information Criterion (AIC) to select the best model using a backward selection approach (smaller is better).

For the statistical analysis of effect of time and hiding box on the body weight, a linear mixed regression model [27] was used to analyse the weight as the outcome and Time after arrival, the availability of a hiding box and the interaction between both as explanatory factors. Although keeping the box in the linear mixed model resulted in a worse fit of the model, the availability of the box nevertheless was added in coherence with our primary aim. Also in this model CatID was used for the random effect.

The validity of both models was confirmed by a visual inspection of the residuals for normality and constance of variance.

Per experimental group the number of adopted cats was analyzed using chi-square test, while the length of stay (LOS) was analyzed using the two-sample T-test. The assumptions for these variables for equal variance (Levene's test) and for normal distribution (Shapiro-Wilk's test) were met.

We reported the estimated effects of the availability of a hiding box according the reporting guidelines for randomized controlled trials (www.reflect-statement.org).

Results

Characteristics of the study population

The experimental group consisted of 12 cats (6 males and 6 females) of which the estimated age ranged between 1 and 7 years (mean: 3.3 years, SD: 2.2). The control group consisted of 11 cats (5 males and 6 females) with estimated ages between 1 to 10 years (mean: 4.9 years, SD: 3.1, with n=10: due to her semi feral behaviour no age could be estimated of cat nr 19).

The cats in this study are presented in the appendix with their ID, experimental group, gender, age, bodyweight at intake (kg) and the quarantine wards they went after intake.

Daily Cat-Stress-Score (CSS): behavioural assessment

The time-dependent reduction of the individual CSS per cat in both groups is visualized in Fig 2.

Fig 2. Course of the Cat-Stress-Score in time of individual cats from the control group and the experimental group.

Cats from the hiding box group reached a steady state sooner (at day 2) than cats from the control group (at day 9). The model results for the mean CSS are presented in Table 1. The estimated means of the CSS of the hiding box group (mean CSS = 2.7) and the control (mean CSS = 3.1) at Day 1 are similar as their difference is not significant (-0.4, 95% CI:-0.97 to +0.12). At all other days the mean CSS of the hiding box group is significantly lower than the mean CSS in the control group, largest at day 2 (-0.99, 95%CI: -1.38 to -0.61) and decreasing in difference between the groups on day 12 (-0.33, 95%CI: -0.57 to -0.08).

Table 1. Results of the model for the Cat-Stress-Score with 95% confidence interval, influenced by Day and availability of a hiding box and interaction between both.

Research group	Time (days after Intake)	Estimate	Lower bound 95% CI[1]	Upper bound 95% CI[1]
Control	Day 1	3.13[2]	2.74	3.53
Control	Day 2	-0.11[3]	-0.43	0.20
Control	Day 3	-0.54[3]	-0.91	-0.17
Control	Day 5	-0.76[3]	-1.15	-0.37
Control	Day 7	-0.82[3]	-1.21	-0.42
Control	Day 9	-0.92[3]	-1.32	-0.53
Control	Day 12	-0.91[3]	-1.34	-0.49
Hiding box	Day 1	-0.43[4]	-0.97	0.12
Hiding box	Day 2	-0.99[4]	-1.38	-0.61
Hiding box	Day 3	-0.51[4]	-0.79	-0.23
Hiding box	Day 5	-0.25[4]	-0.47	-0.03
Hiding box	Day 7	-0.23[4]	-0.40	-0.05
Hiding box	Day 9	-0.12[4]	-0.24	-0.01
Hiding box	Day 12	-0.33[4]	-0.57	-0.08

Legend:
[1] CI = Confidence Interval
[2] Mean CSS in cats in Control group at day 1.
[3] Difference between mean CSS at specified day in Control group compared to mean CSS at day 1 of same cats.
[4] Difference between mean CSS at specified day in cats of group with Hiding box compared to mean CSS of cats in group Control group at same day.

Body weight

For the comparison of both experimental groups, the absolute body weight was used. At intake the control cats were on average 300 grams heavier than those in the experimental group. This initial weight difference between both groups reduced to 210 grams at Day 7 and Day 12: cats in the control group lost overall 7.7% of their initial body weight, while cats with a hiding box lost 6.3% of their initial body weight during those 12 days (Table 2). The initial weight and weight reduction between the groups however proved not to be significant.

Table 2. Results of the model for Body weight with a 95% confidence interval, influenced by Day and availability of a hiding box and interaction between both.

Research group	Time (days after Intake)	Estimate (kg)	Lower bound 95% CI[1] (kg)	Upper bound 95% CI[1] (kg)
Control	Day 0	4,39[2]	3,77	5,01
Control	Day 7	-0,25[3]	-0,35	-0,15
Control	Day 12	-0,32[3]	-0,42	-0,22
Hiding box	Day 0	-0,30[4]	-1,16	0,56
Hiding box	Day 7	-0,21[4]	-1,07	0,65
Hiding box	Day 12	-0,21[4]	-1,07	0,65

Legend:
[1] CI = Confidence Interval
[2] the mean Body weight of cats in the Control group at Day 0.
[3] Difference between the mean Body weight of cats in the Control group at the specified Day compared to the mean Body weight of the same cats at Day 0.
[4] Difference between the mean Body weight of cats in the Hiding box group and the mean body weight of cats in the Control group at the specified Day.

The individual proportional decrease in body weight is visualized in Fig 3. All cats except one lost weight during both weeks. When weight loss at Day 12 was calculated as a percentage of initial body weight at intake, it was found that 7 of the 23 (35%) cats lost ≤ 5% of their body weight, whereas 15 of the 23 (65%) cats lost 5% or more of their weight. The maximum body weight loss was found in cat nr. 8 (control group) which lost 19% of its initial weight in 12 days and was diagnosed with an infection of FeLV a few days after completing this study.

Fig 3. The proportional change (%) in body weight in individual cats from the control group and the experimental group.

Adoption rates and length of stay (LOS)

Of the 23 shelter cats in this study, 21 were rehomed after the observation period was finished. In the control group 9 out of 11 cats were adopted (82%), in the experimental group 12 out of 12 (100%). No significant difference was found in the adoption rate between the two groups (p = 0.55).

As we defined LOS as the number of days between the shelter intake of a cat and its day of adoption, 2 cats were not included in this data set, for they were not adopted.

The mean LOS for the control group (n = 9) was 24.1 days (SD 5.4, range 15-30 days) and for the hiding box group (n= 12) was 22.9 days (SD = 4.4, range 16-30 days). No difference in the mean LOS was found between control and the hiding box group (p-value = 0.58).

Discussion

The aim of the present study was to determine the effect of a hiding box on behavioural stress levels and body weight in shelter cats during the first 12 days in quarantine. While in a previous study cats were monitored on days 1 through 5 and the 14th day [10], this new study added more insight about the differences in CSS between Day 5 and Day 12.

The most important findings of this study are:

- The mean Cat-Stress-Score decreased with time for all cats, but cats with a hiding box however showed a significant faster decrease in the CSS and recovered from stress seven days earlier than the control group.
- Nearly all cats lost significant body weight during the first two weeks. On average, cats with hiding boxes lost 40 grams less of their initial body weight compared with cats without a box, although this difference was not significant.
- The mean adoption rates and the LOS of cats with and without hiding boxes were equal.

Cat-Stress-Score (CSS): behavioural assessment validation

In this study, cats with a hiding box showed a significant faster decrease of behavioral stress compared to the control group, which was most prominent during the first observation days. These results were in line with earlier findings of Vinke et al. [10] and with a study of Gourkow and Fraser, in which the mean CSS of cats, housed in single barren cages without positive human-cat interaction, was higher compared to the other groups and only reached a similar CSS on Day 9 [13].

The findings of the present study complete the results obtained by Vinke et al., where the hiding box group recovered at least four days earlier. By increasing the number of observational days during the first 12 days, the current research provides more details in reaching the CSS-steady state, indicating that hiding boxes accelerate the recovery of behavioral stress by seven days. This is important, because the hiding box clearly helps the shelter cat to adapt more quickly in a stressfull new environment. This in order to prevent the development of chronic stress [17].

Body weight

This study shows a significant decrease in feline body weight during the first 12 days in an animal shelter. Approximately a third of the cats lost less than 5% body weight during the first 12 days, while two-third lost over 5%. These results agree with previous findings of Tanaka et al., in which 57% of their cat population showed 5% or more weight loss during their shelter stay [12].

When otherwise healthy cats loose weight unintentionally, it is a dramatic indicator of a health risk.

Weight loss can be caused by insufficient nutrional management (the shelter offers inadequate quantity and/or quality of food) and also by a decrease in feline appetite by a physical stress response. Although food intake was not registered in the present study, it was observed that some cats were completely anorectic, especially during the first days. For the shelter this was the reason to standardize the feeding schedule of 50 g dry cat food per cat per day. According to the FEDIAF guidelines [24] for daily caloric feline requirements, during this study cats over 4.01 kg might have been offered an inadequate amount of food. With an individual requirement of 80 kcal (335 kJ) ME per $kg^{0.67}$, 50 g dry cat food per day will

meet maintenance energy requirements of cats up to a body weight of 4.01 kg. Cats weighing over 4.01 kg, need more Adult RC food daily. Of the 23 cats, 13 (57%) cats weighed more than 4.01 kg. The heaviest cat weighed 6.41 kg at intake and hence required at least 68.4 grams of catfood per day. During the daily observation however, cats rarely finished their food rations during these first two weeks. An inadequate quantity of food was therefore not considered to be the cause of the observed body weight loss.

The effect of stress however on the body weight of shelter cats was first shown by Tanaka et al. [12], who found a negative correlation between food intake and stress scores of cats. The conclusion was that cats, admitted to an animal shelter were likely to lose weight while in the shelter. These results are consistent with our findings, indicating that a decrease of feline appetite caused by a physical stress response, is most likely responsible for the weight loss.

Although the provided commercial food in this study was of a high quality, there is less understanding of the role of palatability of food for shelter cats in relation to weight loss. The only cat in this study which gained weight, received medication for cystitis (meloxicam 0.05 mg/kg) mixed with canned food. This gives an indication of the importance of palatability of food for shelter cats.

Although the analysis of the effect of time and the presence of a hiding box on the body weight suggested that there was a difference between the two groups in body weight losses, as cats with hiding boxes showed approximately 40 grams less weight loss in comparison with the control group, this difference was not significant. For the individual cat, however, this could be biologically relevant, for weight loss due to feline anorexia has a serious impact on a cat's health, increasing the risks of hepatic steatosis [5,14,15]. A significant difference in body weight between cats with and without hiding facilities, however, was not identified. Therefore more research is necessary to monitor these cats for a longer period of time, to register the process of adaptation to the new environment in correlation to the weight losses and to experiment with ways to prevent or reduce body weight losses in shelter cats (i.e. highly valued palatable food items might overcome the fear motivation and stimulate consumption despite the challenging environment).

Apart from stress, progressive weight loss can also be a sign of serious medical problems [32]. One of the cats from the control group showed a weight loss close to 20% in 12 days and was eventually

diagnosed with FeLV. Shelters could use weight loss during quarantine time as an early warning sign for serious declines in physical conditions, but this asks for a daily weighing as a standard procedure.

Adoption rates and length of stay (LOS)

Sometimes shelter staff expresses their worries about using hiding boxes, for boxes might decrease the visibility of cats to potential adopters and therefore slow down adoption rates (personal communications). Kry and Casey however showed that providing shelter cats with hiding enrichment, did not decrease the likelihood of those cats being adopted [17]. Also our study did not reveal differences in the adoption rates and the LOS of both groups. The hiding enrichment itself, however, could not have influenced the adopters' choices based on the (in)visibility of the cat, for hiding boxes were only present in 12 of the 23 cages during the first 12 days of quarantine time, while no hiding boxes were available in the adoption area.

Finally, Stressors versus Signals of Safety?

Historically, the science of stress emphasizes the role of stressors in evoking stress responses. Stress reduction is caused by reducing the impact of a stressor (in number, strength, duration, etc) on the behavioural and physiological responses of the individual in question by adapting to it [30]. Stressed cats are likely to reduce their feed intake and subsequently loose body weight. Cats in shelters [12], in laboratories [16], in boarding facilities and even privately owned cats [5] show this general stress response to stressors in challenging situations. Because previous research had shown a robust effect of the hiding box on the behavioural stress response [10] reflecting adaption, expectations were that hiding opportunities would also aid in the reduction of weight loss. While this study proved again a significant decrease of the behavioural stress response when shelter cats were offered a hiding opportunity, the effects on body weight were minor. These results challenge our point of focus on stress in shelter animals: a shelter environment offers numerous stressors for which feline hiding behaviour appears not sufficient enough to induce a fast adaptation, for more than 90% of the cats with and without hiding opportunities lost body weight during the first 12 days after intake. New theories on human stress

response mechanisms might shed some light on the feline stress response in these complex shelter environments and contribute to more practical tools for stress reduction. According to Brosschot [33], who introduced the Generalized Unsafety Theory of Stress (GUTS), ‘the stress response of the body is always “on” and it stays on as long as there is no obvious safety.’ This default response can only be inhibited when ‘signals of safety’ are perceived by the animal. We therefore should not look for the causation of a stress response but rather ask ourselves ‘what stops the stress response?’. When present results are reviewed in the light of this GUTS, the hiding enrichment itself caused a decrease in feline behavioural stress scores, but did not provide an adequate signal of safety (SOS) to prevent weight loss in most cats. Changing the food presentation (e.g. food offered inside hiding boxes) might give shelter cats more safety signals. This GUTS approach asks for a comparison of the effect of distinct SOS’s (like hiding materials, food presentations, enriched feeding, feline pheromones, human contact, increased cage space, solitary housing, etc) and for the reinforcing effects of combining these signals on the majority of shelter cats. In addition to focusing on reduction of numerous stressors in the shelter environment, we should also search for signals of safety which are strong enough to inhibit the stress response and thus create a situation which the majority of animals can perceive as safe.

Limitations

Limitations of this randomized controlled trial included the small sample size.

Conclusion

Providing hiding boxes can be a relatively simple way for cats to self-manage stress and to adapt faster to the shelter environment. The majority of the shelter cats however loose (considerable) weight during the quarantine time in an animal shelter. Providing them with hiding enrichment during that period, gives no prevention against this weight loss. Neither do hiding boxes have effect on the adoption rates and the length of stay of both groups.

However, instead of keeping focus on identifying and reducing stressors in a very challenging environment like an animal shelter, an additional approach could be found in the application of ‘signals

of safety' (SOS), strong enough to inhibit the stress response and thus create a situation which animals can perceive as safe.

Acknowledgments

We thank the staff of the animal shelter 'Dierentehuis Arnhem en omstreken' for access to their facilities and their invaluable support during this research. The authors thank the Dutch Society for the Protection of Animals for providing the hiding boxes used in this study.

We wish to thank Prof. Dr. J.W. Hesselink and Dr. R.J. Corbee for their assistance with this study and manuscript preparation.

Author contributions

Van der Leij conceived the study and Vinke assisted with the study design. Selman assisted with data collection and data maintenance. Vernooij, Selman and Van der Leij analyzed the data and drafted the manuscript. Vinke supervised the draft and submission.

Conflicts of interest

The authors declare that no conflict of interest exists in which any author or authors' institution has a financial or other relationship with other people or organizations that may inappropriately influence the authors' work.

References

[1] HAS Hogeschool & Utrecht University faculty of Veterinary medicine. 2015. *Feiten & Cijfers Gezelschapsdierensector 2015*.

[2] Bradshaw, J. W. S., Rachel A. Casey, and Sarah L. Brown. 2012. *The Behaviour of the Domestic Cat.* 2nd ed. Wallingford: CABI.

[3] McCobb, E. C., G. J. Patronek, A. Marder, J. D. Dinnage, and M. S. Stone. 2005. "Assessment of Stress Levels among Cats in Four Animal Shelters." *Journal of the American Veterinary Medical Association* 226 (4): 548-555. doi:10.2460/javma.2005.226.548.

[4] Newbury, S., M. K. Blinn, P. A. Bushby, C. B. Cox, J. D. Dinnage, B. Griffin, K. F. Hurley, et al. 2010. *Guidelines for Standards of Care in Animal Shelters*: The Association of Shelter Veterinarians.

[5] Amat, M., T. Camps, and X. Manteca. 2016. "Stress in Owned Cats: Behavioural Changes and Welfare Implications." *Journal of Feline Medicine and Surgery* 18 (8): 577-586. doi:10.1177/1098612X15590867.

[6] Sabioncello, A., K. Gotovac, A. Vidovic, A. Gagro, A. Markotic, S. Rabatic, and D. Dekaris. 2004. "The Immune System Under Stress." *Periodicum Biologorum* 106 (4): 317-323.

[7] Moberg, Gary and Joy A. Mench. 2000. *The Biology of Animal Stress Basic Principles and Implications for Animal Welfare*. Oxon, UK; New York: CABI.

[8] Griffin, J. F. T. 1989. "Stress and Immunity: A Unifying Concept." *Veterinary Immunology and Immunopathology* 20 (3): 263-312. doi:10.1016/0165-2427(89)90005-6.

[9] Pesavento, P. A. and B. G. Murphy. 2014. "Common and Emerging Infectious Diseases in the Animal Shelter." *Veterinary Pathology* 51 (2): 478-491. doi:10.1177/0300985813511129.

[10] Vinke, C. M., L. M. Godijn, and W. J. R. van der Leij. 2014. "Will a Hiding Box Provide Stress Reduction for Shelter Cats?" *Applied Animal Behaviour Science* 160 (1): 86-93. doi:10.1016/j.applanim.2014.09.002.

[11] Ottway, D. S. and D. M. Hawkins. 2003. "Cat Housing in Rescue Shelters: A Welfare Comparison between Communal and Discrete-Unit Housing." *Animal Welfare* 12 (2): 173-189.

[12] Tanaka, A., D. C. Wagner, P. H. Kass, and K. F. Hurley. 2012. "Associations among Weight Loss, Stress, and Upper Respiratory Tract Infection in Shelter Cats." *Journal of the American Veterinary Medical Association* 240 (5): 570-576. doi:10.2460/javma.240.5.570.

[13] Gourkow, N. and D. Fraser. 2006. "The Effect of Housing and Handling Practices on the Welfare, Behaviour and Selection of Domestic Cats (Felis Sylvestris Catus) by Adopters in an Animal Shelter." *Animal Welfare* 15 (4): 371-377.

[14] Hall, J. A., L. A. Barstad, and W. E. Connor. 1997. "Lipid Composition of Hepatic and Adipose Tissues from Normal Cats and from Cats with Idiopathic Hepatic Lipidosis." *Journal of Veterinary Internal Medicine / American College of Veterinary Internal Medicine* 11 (4): 238-242. doi:10.1111/j.1939-1676.1997.tb00097.x.

[15] Biourge, V. C., J. M. Groff, R. J. Munn, C. A. Kirk, T. G. Nyland, V. A. Madeiros, J. G. Morris, and Q. R. Rogers. 1994. "Experimental Induction of Hepatic Lipidosis in Cats." *American Journal of Veterinary Research* 55 (9): 1291-1302.

[16] Carlstead, K., J. L. Brown, and W. Strawn. 1993. "Behavioral and Physiological Correlates of Stress in Laboratory Cats." *Applied Animal Behaviour Science* 38 (2): 143-158. doi:10.1016/0168-1591(93)90062-T.

[17] Kry, K. and R. Casey. 2007. "The Effect of Hiding Enrichment on Stress Levels and Behaviour of Domestic Cats (Felis Sylvestris Catus) in a Shelter Setting and the Implications for Adoption Potential." *Animal Welfare* 16 (3): 375-383.

[18] Kessler, M. R. and D. C. Turner. 1997. "Stress and Adaptation of Cats (Felis Silvestris Catus) Housed Singly, in Pairs and in Groups in Boarding Catteries." *Animal Welfare* 6 (3): 243-254.

[19] Nederlandse overheid. "Artikel 8 Lid 3 Burgerlijk Wetboek Nr 5 ." Available from: https://wetten.overheid.nl/BWBR0005288/2018-09-19 , accessed 03-22, 2019.

[20] Gunn-Moore, D., K. Moffat, L. -A Christie, and E. Head. 2007. "Cognitive Dysfunction and the Neurobiology of Ageing in Cats." *Journal of Small Animal Practice* 48 (10): 546-553. doi:10.1111/j.1748-5827.2007.00386.x.

[21] UC Davis University Koret Shelter Medicine Program. "Spot Cleaning Cat Cages.", available from: https://www.sheltermedicine.com/library/resources/?utf8=%E2%9C%93&site=sheltermedicine&search%5Bslug%5D=spot-cleaning-cat-cages , accessed 03-22, 2019.

[22] UK Cat Behaviour Working Group. 1995. *An Ethogram for Behavioural Studies of the Domestic Cat (Felis Silvestris Catus L.).* UK: UFAW Animal Welfare Research Report 8. Universities Federation for Animal Welfare, UK.

[23] Lehner, P. N. 1992. "Sampling Methods in Behavior Research." *Poultry Science* 71 (4): 643-649. doi:10.3382/ps.0710643.

[24] FEDIAF. "Nutritional Guidelines for Complete and Complementary Pet Food for Cats and Dogs .", available from: http://www.fediaf.org/images/FEDIAF_Nutritional_Guidelines_Update_December_2018.pdf , accessed 03-27, 2019.

[25] Pfeiffer, D. 2010. "Chapter 4: Designing Epidemiological Studies. ." In *Veterinary Epidemiology: An Introduction.* 1st ed., 33-42. Chichester, U.K.; Ames, Iowa: Wiley-Blackwell.

[26] Ellis, S. L. H., I. Rodan, H. C. Carney, S. Heath, I. Rochlitz, L. D. Shearburn, E. Sundahl, and J. L. Westropp. 2013. "AAFP and ISFM Feline Environmental Needs Guidelines." *Journal of Feline Medicine and Surgery* 15 (3): 219-230. doi:10.1177/1098612X13477537.

[27] Pinheiro, J., Bates, D., DebRoy, S., Sarkar, D. and R Core Team. "Nlme: Linear and Nonlinear Mixed Effects Models.", available from: https://cran.r-project.org/web/packages/nlme/index.html , accessed 03-27, 2019.

[28] R Foundation for Statistical Computing. "R: A Language and Environment for Statistical Computing.", available from: https://www.R-project.org/ , accessed 03-22, 2019.

[29] Suresh, K. 2011. "An Overview of Randomization Techniques: An Unbiased Assessment of Outcome in Clinical Research." *Journal of Human Reproductive Sciences* 4 (1): 8-11. doi:10.4103/0974-1208.82352.

[30] Logan, J. G. and D. J. Barksdale. 2008. "Allostasis and Allostatic Load: Expanding the Discourse on Stress and Cardiovascular Disease." *Journal of Clinical Nursing* 17 (7B): 201-208. doi:10.1111/j.1365-2702.2008.02347.x.

[31] Bestuur Dierentehuis De Dierenborgh. 2014. *Jaarverslag 2013*. Velp: Dierentehuis De Dierenborgh.

[32] Hartke, J. R., K. A. Hayes, C. A. Buffington, L. E. Mathes, and J. L. Rojko. 1995. "Acute Feline Leukemia Virus Infection Causes Altered Energy Balance and Growth Inhibition in Weanling Cats." *Journal of Acquired Immune Deficiency Syndromes and Human Retrovirology* 9 (1): 11-19.

[33] Brosschot, J. F., B. Verkuil, and J. F. Thayer. 2018. "Generalized Unsafety Theory of Stress: Unsafe Environments and Conditions, and the Default Stress Response." *International Journal of Environmental Research and Public Health* 15 (3). doi:10.3390/ijerph15030464.

[34] Sargeant, J. M., A. M. O'Connor, I. A. Gardner, J. S. Dickson, M. E. Torrence, I. R. Dohoo, S. L. Lefebvre, P. S. Morley, A. Ramirez, and K. Snedeker. 2010. "The Reflect Statement: Reporting Guidelines for Randomized Controlled Trials in Livestock and Food Safety: Explanation and Elaboration." *Zoonoses and Public Health* 57 (2): 105-136. doi:10.1111/j.1863-2378.2009.01312.x.

Supporting information

S1 Fig 1. Diagram of the experimental set up, observer and both camera positions in the quarantine wards in the animal shelter.

S2 Fig 2. Course of the Cat-Stress-Score in time of individual cats from the control group and the experimental group.

S3 Fig 3. The proportional change (%) in body weight in individual cats from the control group and the experimental group.

S1 Appendix. Baseline characteristics of treatment cohorts in a randomized field trial comparing quarantine cat housing with and without hiding opportunities in one Dutch animal shelter.

Figure

Click here to download Figure S1_Fig 1 - Camera position.bmp

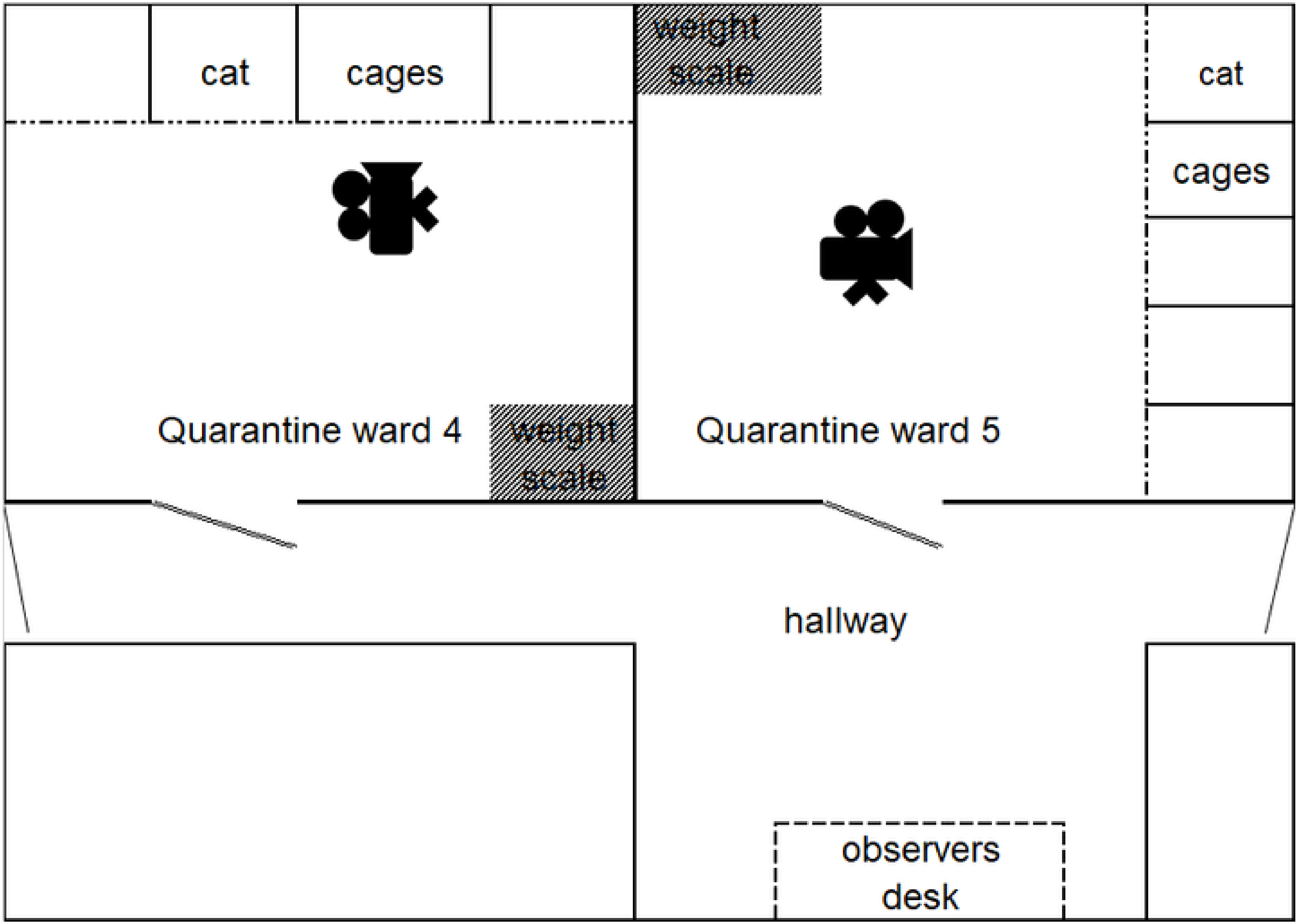

Figure

Click here to download Figure S2_Fig 2 - CSS individual.bmp

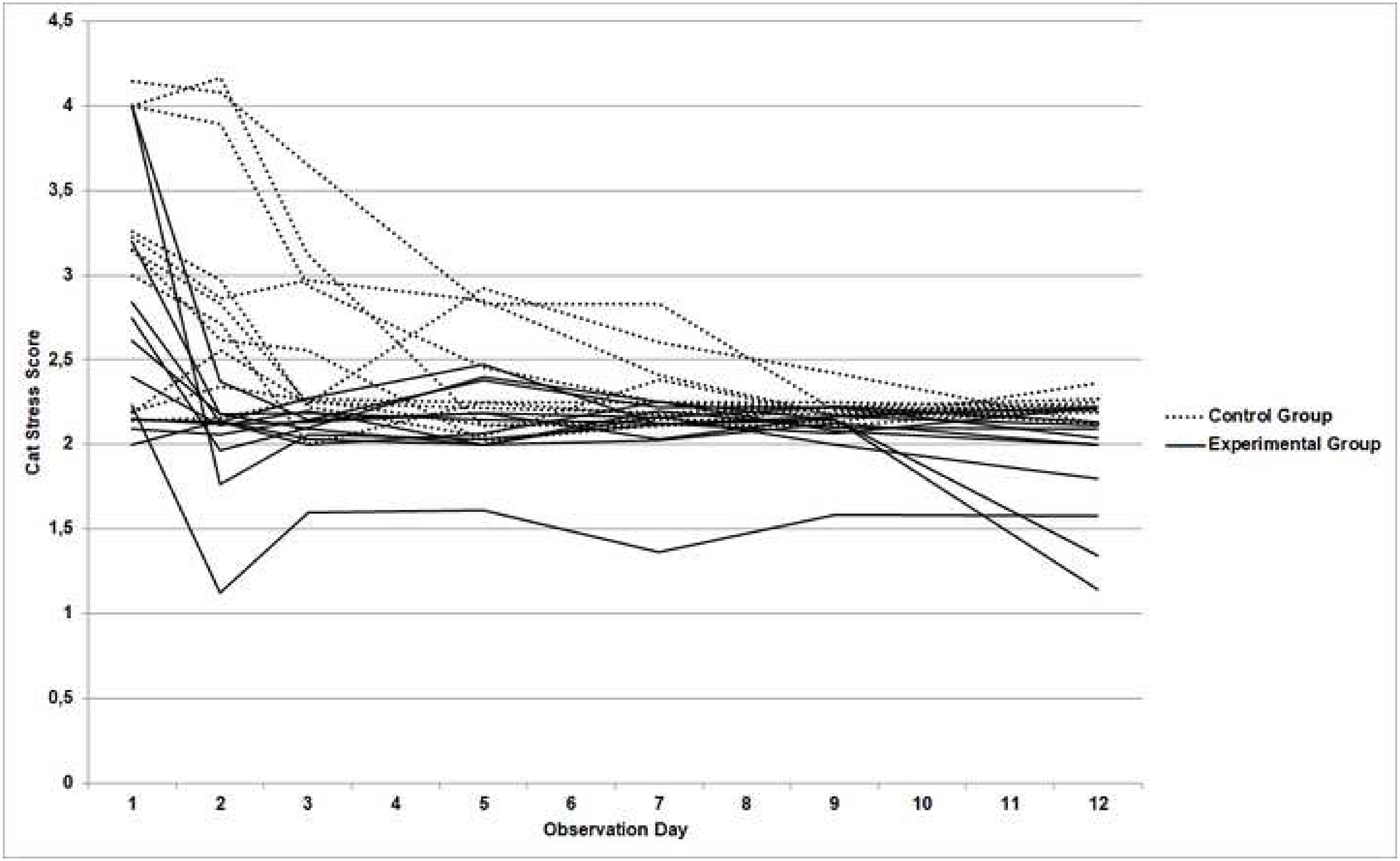

Figure

Click here to download Figure S3_Fig 3 - Body Weight individual.bmp

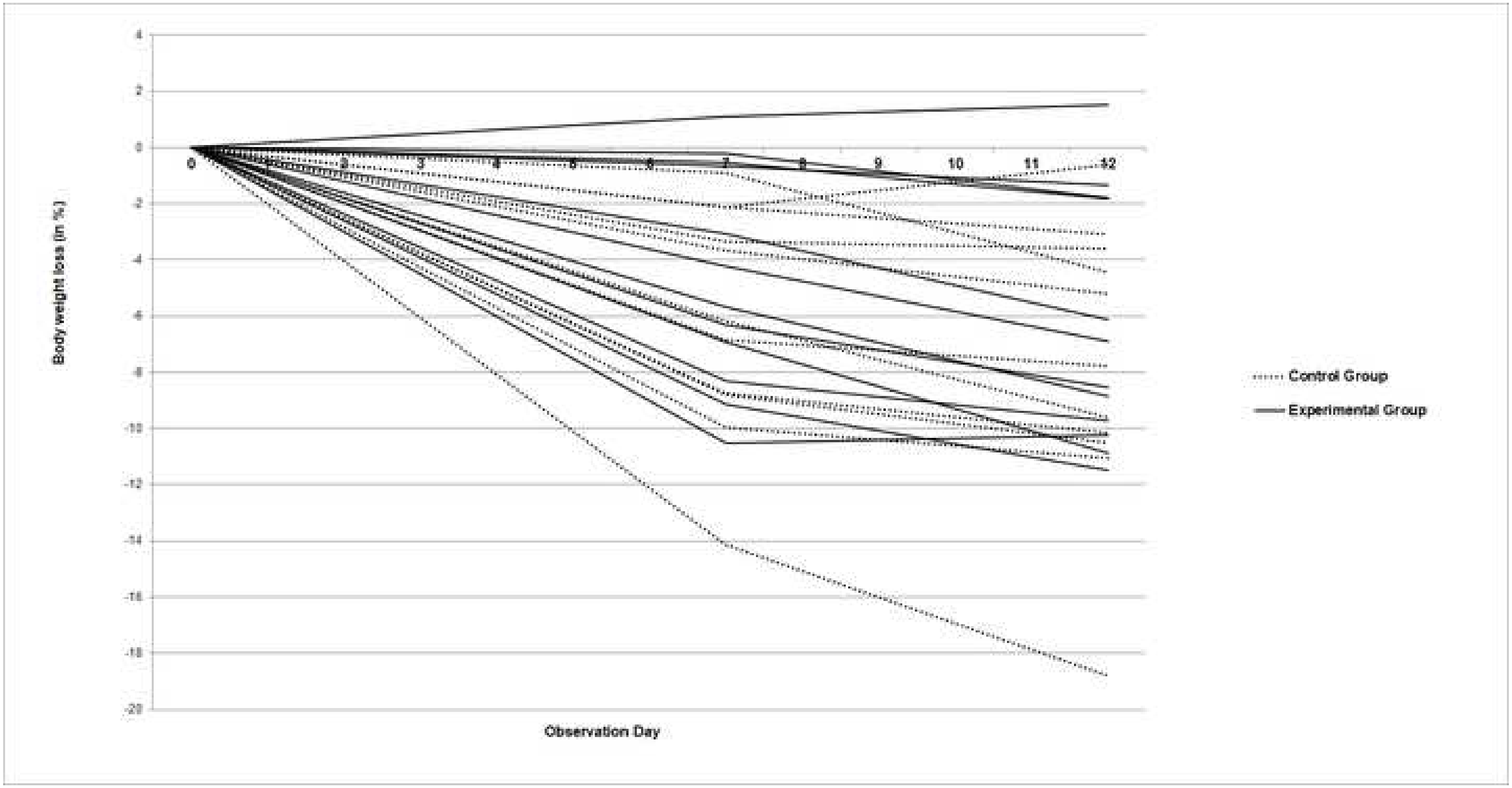

Click here to access/download
Supporting Information
S1_Appendix base line.bmp
