# Supplementary material for: The effect of a hiding box on stress levels and body weight in Dutch shelter cats; a randomized controlled trial
Source: PLoS One. 2019 Oct 14;14(10):e0223492. doi: 10.1371/journal.pone.0223492 (PMC6791553; doi:10.1371/journal.pone.0223492)
Supplement: Attachment — Submitted filename: Response to reviewers PONE-D-19-15024 - 27 aug 2019.docx [file pone.0223492.s004.docx]

Response to reviewers - manuscript entitled “The effect of a hiding box on stress levels and body weight in Dutch shelter cats; a randomized controlled trial”, PONE-D-19-15024

We thank the reviewers for their generous comments and efforts towards improving our manuscript.

Using their comments, we corrected several crucial aspects of our manuscript:

- Figure 1 and 2 were redrawn and improved information about the graphs of individual changes in CSS and body weight per cat was added.
- The importance of a minor size deviation between individual cat cages was addressed, based on recent studies.
- Differences between acute and chronic stress were pointed out.
- The section ‘Limitations’ of this study was completely revised.

In this rebuttal letter we like to respond to the points raised by the academic editor and reviewers. Our comments are combined in a general response to our reviewers, while specific comments are addressed to each reviewer.

Points raised by reviewers are written in black *Italic texts*, our response is written in green text.

Lines mentioned by the authors are referring to the revised version of the manuscript.

Response to Reviewers on general comments

- **Grammar and use of English language**

Reviewer #1:

- Lines 37-38: *Grammar suggestion "...but cats with a hiding box showed a significantly faster..."*
- Line 101: *Spelling "vaccine"*
- Line 110: *Grammar suggestion "Two cats participating in this study left..."*

Reviewer #3

- Line 66: *The reference number for Vinke et al., is missing.*
- Line 102*: ‘day 14’ not ‘Day 14’.*
- Line 213: *What did you mean when you used the word ‘constance’? is this a typo?*
- Line 298: *please delete the word ‘however.’*
- Line 79: *Because the Ethics committee approved the study, then please provide the protocol number.*
- Lines 120-122: *The structure of this sentence is unclear. It needs to be rewritten.*

Reviewer #4:

- *Review the manuscript with a close eye to correct choice of words - getting it proofread by a third party may be helpful here. Examples include e.g. loose instead of lose; expresses instead of expressed.*

Response of the authors:

Our thanks to all reviewers for their careful reading of our manuscript and addressing these aspects. The errors were corrected and the revised manuscript was proofread by a British colleague.

- **Shortening of Discussion**

Reviewer #1:

- *Shorten the discussion. A great deal of information provided is repetitious. Clarify what is known and what is presumed/thought/supposed.*

Response of the authors:

Thank you for pointing this out. We have clarified, shortened and excluded several repetitive texts from the discussion.

Response addressed to the individual reviewer

**Reviewer #1:**

1 - *It is not clear that enough animals were evaluated or that the amount of food ingested by each cat was measured. It was clear that not all cats had the same diet (one cat that gained weight was given wet food).*

7 - Lines 230-183: *Report what the individual cats ate. Did they all finish the food they were given each day?*

Response of the authors:

We agree on this comment and apologize for the uncertainties in the text.

The corrected text in ‘Material and methods’ (lines 190 – 193):

All cats were offered the same diet, with some individual temporal exceptions which are mentioned in the text (oral medication would be given mixed with tinned food). The shelter feeding regime did not include the monitoring of the daily intake of food per cat and neither did our study design.

The corrected text in ‘Discussion - Limitations’ (lines 478 – 481):

During this research, feeding protocols, based on long-term experience of the shelter staff, were unaltered. By doing so this study created a better understanding of the effect of shelter feline care on behavioral parameters and body weight of these cats. For subsequent research, however, it is recommended to gather data on the daily intake of food and water per individual shelter cat.

2 - *The length of stay of the cats was compared. However, hiding boxes were not used in the adoption area. So the reasoning for how hiding boxes affect length of stay is not clear. Did the authors predict that the cats would be less social when they were removed from the "safety" of the box and transferred to the adoption area?*

Response of the authors:

We agree on this comment, the text has not been clear about this and this is corrected.

The corrected text in ‘Discussion – Length of stay (LOS)’ (lines 418 – 429):

Providing cats with hiding enrichment at any stage of their shelter stay requires an investment in shelters’ scarce time and money. Shelter staff sometime have their reservations about using hiding boxes, for it might decrease the visibility of cats to potential adopters and therefore slow down adoption rates (personal communications). Although Kry and Casey found no significant difference in the Length of Stay between cats with and without hiding boxes in the adoption ward [17], in the present study the hiding enrichment itself could not have influenced the adopters’ choices based on the (in)visibility of the cat. Hiding boxes were only present in 12 of the 23 cages during the first 12 days of quarantine time, while no hiding boxes were available in the adoption area.

Our study showed a decrease in the CSS during quarantine time, while eventually both groups had similar rates for adoption and LOS. In sum, providing hiding enrichment to shelter cats benefits the welfare of the animals without having negative consequences for the shelter (like an increased LOS per cat).

3 - Specific suggestions: *Address the concern of low power (low N) for the statistics involving weight loss.*

Response of the authors:

The reviewer is right, more cats in our study would have been preferable. However, research in a shelter setting presents itself with challenges, like the number of cats available during a certain time. However, based on the acquired data we feel no hesitations in expressing our doubts about the preventive effect of a hiding box on the loss of body weight.

The corrected text in ‘Discussion - Limitations’ (lines 470 – 472, 474 – 476):

Conducting the present randomized controlled trial research in an operating animal shelter comes with its inherent limitations. Restrictions in research time consequently affects the number of animals available [ ].

This study is based on a small sample of shelter cats and only captures the first 12 days after intake. The evaluation of stress impacting the body weight of cats likely requires a longer assessment period and a larger dataset to be able to apply these insights to a wider cat population in shelters. Despite these limitations, the current findings provide evidence that hiding boxes may not be able to prevent significantly weight loss in shelter cats and are instructive for our understanding of the effects of hiding enrichment on the feline behavioural parameters.

4 - *Figure 1 is unnecessary.*

Response of the authors:

Thank you, we added this figure to the Appendix section.

5 - Lines 187-189. *Clarify the calculation of LOS. Which cats? All cats in the shelter? Only cats in the study?*

Response of the authors:

We apologize for not being clear in these aspects.

The corrected text in ‘Material and methods – Length of stay (LOS)’ (lines 200 – 201):

LOS: number of days between the shelter intake of the cats in this study and their day of adoption.

6 - Lines 222-228: *The difference observed in the initial average weight of the groups would be beneficial to include here.*

Response of the authors:

You are correct and this was included in this section.

The corrected text in ‘Results - Characteristics of the study population’ (lines 238 – 239):

At intake, the control cats were on average 300 grams heavier than those in the experimental group.

8 - *Measurement of a specific biochemical marker of stress would have been helpful to report.*

Response of the authors:

Yes, we agree on this comment. In our next studies feline cortisol is included (not published yet). We added a comment to the limitation section.

The corrected text in ‘Discussion - Limitations’ (lines 482 – 485):

The behavioural stress parameter of the Cat Stress Score was combined with the physical parameter of body weight. Although stress has previously been shown to negatively affect feline body weight [12], the inclusion of a specific biochemical marker of stress would have strengthened the outcomes of this study. Preferably both physical and biochemical feline stress markers are combined in future studies.

**Reviewer #2:**

1 - *The housing used for cats in the study was not the same across the 2 groups. Housing may affect stress levels in individual cats and the differences in the size of the housing used looks to be significant - one cage type provided 1221cm squared of floor space and the other was 945cm squared of floor space. It was not indicated that this was controlled for in the study. Of the 12 cats housed with hiding structures 10 were in the larger housing units. The control group was more evenly distributed between small and larger cages with 6 controls in the smaller cage and 5 in the bigger cage. I am concerned this is confounding variable in this study and should be controlled for.*

Response of the authors:

Thank you for mentioning it, for this aspect needs more clarification.

Between the two cat cages we used in our study, the difference was around a 20% difference in floor space. In previous publications Kessler et. al. (1999) had observed:

“Although these results indicate that cage size has an effect on stress in cats housed singly, they give no indication of the minimum cage size required to reduce that stress. It has also been suggested that certain qualitative aspects of the housing environment may have an important influence on reducing stress among singly housed animals”.

More recent studies [Stella 2017] proved him right: even doubling the floor space per cat did not result in differences in behavior between cats. Cats seem to be less sensitive for space requirements, but far more sensitive to other qualities like environmental enrichments and quality of care. We therefore added more information to the Materials and methods and to the general discussion.

The corrected text in ‘Material and methods – Housing conditions’ (lines 136 – 138):

The cat housing in the two adjacent quarantine wards consisted of cages (L x W x H: either 84 x 95 x 80 cm with elevated perching shelves (at height of 28 cm) of 84 x 25 cm or 69 x 91 x 87 cm with shelves (equal height) of 69 x 25 cm) in which the cats were individually housed. Available floorspace per cage (cage floor plus perching shelf) was respectively 1.01 m^2^ and 0.80 m^2^.

The corrected text in ‘Discussion’ (lines 346 – 354):

The cages used for the cats in this experiment, varied in dimensions: the smaller cage offered 80% of the floor space of the larger one. Although Kessler and Turner [41] showed an effect of cage dimensions (0.49 m^2^ versus 1.02 m^2^) on stress levels in individual cats, they considered other qualitative aspects of the housing environment to be of importance as well. More recent research [38] studied the effect of cage dimensions (1.1 m^2^ versus 0.56 m^2^) combined with different feline care regimes. Doubling the cage sizes did not influence the pattern of feline responses, suggesting that physical space solely may be of less importance to cats, in contrast to managed and enriched environments that did significantly affect the cats. Therefore, in this present study, the effects of hiding enrichment were studied in spite of a minor size deviation between the individual cat cages.

**Reviewer #3:**

1 - Lines 50-54: *What is the definition of ‘chronic’? I think it will benefit your manuscript if you clearly define a ‘window of time’ that directly correlates with well-defined clinical outcomes.*

Response of the authors:

Yes indeed, a comparison between ‘chronic stress’ and ‘acute stress’ is needed. A time frame however, does not adequately define these differences in stress (like: acute stress = stress endured during a short period of time, chronic stress = stress endured during a prolonged period). Most stress research focuses therefore on the effects of stress on physical systems: whether or not stress is harmful to these systems. Acute stress induces adaptation (no physical harm) while chronic stress dysregulate several physical systems (and therefore induces harm). The moment of dysregulation per system differs in time and makes it difficult to generally time chronic stress. We therefore chose to make a distinction between ‘acute’ and ‘chronic’ stress by emphasizing the differences in harmful effects. Adaptation caused by acute stress might be visualised by a change in CSS, while chronic stress might result in the observed body weight loss.

The corrected text in ‘Introduction’ (lines 51 – 57):

Acute stress will encourage the animal to adapt to its surroundings. However, when prolonged aversive stimuli interfere with the adaptation ability, chronic stress will develop by causing a dysregulation of several major physiological systems like the hypothalamic–pituitary–adrenal (HPA) axis. This may elicit clinical signs, such as hiding behaviour, defecating and urinating outside the litter box, decreased grooming or over-grooming behaviour and a loss of appetite [2,5-8]. Stress-induced long-term high cortisol levels can reduce the efficacy of the immune system against infectious diseases [1,5,6,8,9,42], and chronic stress can therefore harm a cat’s health as well [5,7,10,11].

*4 - Line 124: Your manuscript will benefit from clearly defining the method of randomization. Was according to an online randomization tool? Coin toss? Etc.*

Response of the authors:

Thank you for mentioning this omission. We used a webbased randomization tool, which was added to the manuscript.

The corrected text in ‘Materials and methods - Animals’ (line 133):

The 23 cats were randomly assigned to one of the two groups with and without access to a hiding box, using an online randomization tool [39].

The corrected text in ‘References’ (lines 632 – 633):

[39] "Research Randomizer" by the Social Psychology Network. Available from: <https://www.randomizer.org/> , accessed 10-25, 2015.

5 - Line 138: *Do you think that the shelter in this study represents the setting in which many other shelters in the Netherlands and worldwide are set-up? In other words, do you know whether many / most other places have cats mixed with dogs and other stressors such as large traffic within the shelter that were not present in this shelter? You are attempting to test a method for stress prevention/reduction which is great, but if the setting of the study may not represent many other facilities, then the external validity of this study is in question. I am writing this from the standpoint of a reviewer who supports the publication of this study, so I am not looking for a defensive response… I am suggesting that you carefully think about this point and that in the revised manuscript you will discuss it and also point out how the results of this study could be leveraged to promote new regulation for shelter management and could be used as a stepping board for upcoming studies.*

Response of the authors:

Thank you for your kind words and concern.

When looking for a research site, this was one of our requirements: to find an average animal shelter, which was not particularly large or providing exceptionally good care or even suboptimal care, and was willing to house our team. The animal shelter which allowed us to use their facility is exemplary for the older, medium size Dutch animal shelter. The majority of the 115 official Dutch shelters are built in the ‘70’s according to the legal standards at that time. Without exception cats and dogs are housed in the same shelter facility, separated in different wards. Indirectly they mix through occasional visual contact, sounds and smell. Only recently new Shelter Medicine insights have initiated gradual change in the care for shelter animals. Although our study in 2014 (Vinke et. al) about the effect of hiding boxes for shelter cats for instance stimulated the use of hiding boxes for cats in Dutch shelters, shelter housing of cats and dogs has not changed fundamentally.

The corrected text in ‘Materials and methods – Animal shelter’ (lines 97 – 98):

The housing of the animals, their care and management provided by the shelter in this study is representative of the majority of Dutch shelters.

6 - Lines 150-153: *Are there any previous studies that indicated that the introduction of a new static object in front of a cat cage is not a stressful situation by itself. If there are studies that have shown it is not, then please cite them. Otherwise, in the limitation paragraph among the other things that I pointed out, you will need to discuss the possibility that mounting a camera in front of the cage could have induced stress and in theory, could be partially the reason for why there was no significant difference in body weight between the groups. Also, if it was not explored before, you should mention that it is something that needs to be studied in the future.*

7 - Line 170-171: *This is related to my comment for lines 150-153; how do we know that 2 minutes is enough time. How do you know that it does not lead to prolonged stress…? If there is previous research to support that it is not, then cite it and it will make your study stronger. If you do not know, you will need to discuss it under the limitation paragraph.*

Response of the authors:

Very true indeed. As far as we know no research has been published on this aspect. We therefore mentioned it in the section Limitations.

The corrected text in ‘Limitations’ (lines 486 – 491):

The shelter cats were observed with a video camera mounted in front of their cages, a technique used in several studies [35,36,37,38]. In the previous [10] and current study cats were given a habituation time to positioning the camera of 2 minutes, before starting the observation. To the authors’ knowledge, the stress inducing effect of placing a novel static object in front of a cat cage has never been studied in cats. Its effect on the loss of body weight in both groups cannot be ruled out and therefore new research is recommended to investigate the stress inducing effect of this observation method in shelter cats.

8 - Figure 2: *I strongly suggest that you will consider plotting the figure as a series of grouped boxplots with the CSS on the y-axis and the days it was assessed on the x-axis. Firstly, I think that it will be clearer than a bunch of lines that intersect and overlap with each other. Secondly, the CSS is not a continuum and therefore should not have a connecting line. For example, you do not know if there wasn’t a peak of stress in one of the days when it was not evaluated or during other hours on the days that you did…*

11 - Figure 3: *I strongly suggest that you will consider plotting the figure as a series of grouped boxplots with the %change in body weight on the y-axis and the days it was assessed on the x-axis — same reasons as for figure 1.*

Response of the authors:

Thank you for mentioning it, this aspect needs more clarification as it otherwise might cause some misunderstanding. The connecting lines in fig 1 and 2 do not suggest a linearity of the measured parameters (CSS/loss of body weight (BW)) between two measurements, but the course of change in CSS/BW per individual cat in time. When these data would be plotted as grouped boxplots, this distinct individual information would be lost.

To clearify this I have adjusted the figures by adding the time points of measurements per individual cat and added information to the legenda as well.

The corrected text in ‘Fig 1 – legenda’ (lines 247 – 249):

Line segments connect measurements within the same cat to show the change of CSS in course of time. Dotted lines: individual cats without hiding boxes (control group). Solid lines: individual cats with hiding box (experimental group).

The corrected text in ‘Fig 2 – legenda’ (lines 317 – 320 ):

Line segments connect measurements within the same cat to show the change of body weight in course of time. Dotted lines: individual cats without hiding boxes (control group). Solid lines: individual cats with hiding box (experimental group).

9 - Table 1: *I strongly suggest that you will consider reconstructing your table such that the left column will have the days; the next column will have the control mean (95CI), and in the last column the hiding box mean (95CI).*

10 - Table 2: *I strongly suggest that you will consider reconstructing your table such that the left column will have the days; the next column will have the control mean BW change from baseline (95CI) and in the last column the hiding box mean BW change from baseline (95CI). It would be much more meaningful for the reader to know the %change in BW from baseline. You can rerun your regression by incorporating it into your data file as a new column with the percent change for days 7 and 12 relative to baseline.*

Response of the authors:

Thank you, the tables are corrected according to your valuable suggestions!

12 - Line 403: *the limitation paragraph should include the limitations I have discussed throughout my reviewer response. In addition, you need to address the limitation that the CSS in this study was not coupled with biomarkers of stress, for example, cortisol.*

Response of the authors:

Yes, we agree on this comment, given also by reviewer 1. In our next studies feline cortisol is included (not published yet). We added this comment to the limitation section.

The corrected text in ‘Discussion - Limitations’ (lines 482 – 485):

The behavioural stress parameter of the Cat Stress Score was combined with the physical parameter of body weight. Although stress has previously been shown to negatively affect feline body weight [12], the inclusion of a specific biochemical marker of stress would have strengthened the outcomes of this study. Preferably both physical and biochemical feline stress markers are combined in future studies.
